# Serum dickkopf-3 is associated with death and vascular events after ischemic stroke: an observational study from CATIS

**DOI:** 10.1186/s12974-019-1680-4

**Published:** 2020-01-09

**Authors:** Zhengbao Zhu, Daoxia Guo, Chongke Zhong, Aili Wang, Tan Xu, Yanbo Peng, Hao Peng, Qunwei Li, Zhong Ju, Deqin Geng, Jing Chen, Yonghong Zhang, Jiang He

**Affiliations:** 1 Department of Epidemiology, School of Public Health and Jiangsu Key Laboratory of Preventive and Translational Medicine for Geriatric Diseases, Medical College of Soochow University, 199 Renai Road, Industrial Park District, Suzhou, Jiangsu Province, 215123 China; 2 Department of Epidemiology, School of Public Health and Tropical Medicine, Tulane University, New Orleans, LA USA; 3 Department of Neurology, Affiliated Hospital of North China University of Science and Technology, Tangshan, Hebei China; 4 Department of Epidemiology, School of Public Health, Taishan Medical College, Tai’an, Shandong China; 5 Department of Neurology, Kerqin District First People’s Hospital of Tongliao City, Tongliao City, Inner Mongolia China; 6 Department of Neurology, Affiliated Hospital of Xuzhou Medical University, Xuzhou, Jiangsu China; 7 Department of Medicine, School of Medicine, Tulane University, New Orleans, LA USA

**Keywords:** Dickkopf-3, Ischemic stroke, Biomarkers, Prognosis, death, Vascular events

## Abstract

**Background:**

Dickkopf-3 (Dkk-3) is implicated in the progression of atherosclerosis. This study aimed to investigate the association between serum Dkk-3 and the prognosis of ischemic stroke.

**Methods:**

We measured serum Dkk-3 levels in 3344 ischemic stroke patients from CATIS (China Antihypertensive Trial in Acute Ischemic Stroke). The primary outcome was a combination of death and vascular events within 3 months after ischemic stroke.

**Results:**

During 3 months of follow-up, the cumulative incidence rates of primary outcome among ischemic stroke patients in five quintiles of serum Dkk-3 (from low to high) were 4.49%, 3.74%, 2.54%, 5.23%, and 6.73%, respectively (log-rank *p* = 0.004). Multivariable Cox proportional hazards regression analyses showed that compared with the third quintile of serum Dkk-3, the adjusted hazard ratios (95% confidence intervals) associated with the first and fifth quintile were 3.49 (1.46–8.34) and 4.23 (1.86–9.64) for primary outcome, 3.47 (1.06–11.36) and 5.30 (1.81–15.51) for death, and 2.66 (1.01–7.01) and 3.35 (1.33–8.40) for vascular events, respectively. Multivariable-adjusted Cox proportional hazards regression model with restricted cubic splines showed a U-shaped association between serum Dkk-3 and the risk of primary outcome (*p* for nonlinearity = 0.030). Moreover, adding serum Dkk-3 to conventional risk factors could improve the predictive power for primary outcome (net reclassification improvement 28.44%, *p* < 0.001; integrated discrimination improvement 0.48%, *p* = 0.001).

**Conclusions:**

Both low and high serum Dkk-3 levels are associated with increased risks of death and vascular events within 3 months after ischemic stroke, indicating that serum Dkk-3 may have a special effect on the prognosis of ischemic stroke. We also found that serum Dkk-3 might be a prognostic biomarker for ischemic stroke. Further studies are needed to replicate our findings and to determine the optimal levels of serum Dkk-3.

## Background

Stroke is the second most common cause of death and the leading cause of long-term disability worldwide [1]. In China, there are 2.5 million new stroke cases each year and more than one fifth of deaths are attributed to stroke, which leads to an enormous economic burden to the families and society as whole [2]. The most common type of stroke is ischemic stroke and accounts for approximately 78% of all stroke cases [3]. The development of adverse clinical outcomes after ischemic stroke is complex and may involve many factors including older age, neurological impairment, diabetes, hypertension, and dyslipidemia [4, 5]. However, all these established traditional risk factors can only explain part of poor prognosis of ischemic stroke [5]. Therefore, identifying novel biomarkers associated with poor prognosis will assist the selection of patients at high risk for aggressive monitoring and therapeutic interventions.

Dickkopf-3 (Dkk-3) is a secreted protein of Dkk family involving in regulating Wnt pathway and determining cell fate during embryonic development [6, 7]. Some studies showed that Dkk-3 could play important roles in the development and progression of atherosclerosis [8, 9]. Increased Dkk-3 expression accelerated the atherosclerotic process and promoted plaque accumulation, and reduction of Dkk-3 could decrease the size of atherosclerotic lesions in ApoE-deficient mice [8]. Conversely, as an important regulator of cell fate determination [10, 11], Dkk-3 was also reported to have certain protective roles against atherosclerosis. Plasma Dkk-3 concentration was inversely related to carotid artery intima-media thickness, and Dkk-3 could promote re-endothelialization after atherosclerosis or artery injury [9].

Given the important regulating roles of Dkk-3 in the process of atherosclerosis, it is of clinical interest to investigate whether serum Dkk-3 can provide additional prognostic value for ischemic stroke. However, the relationship between serum Dkk-3 and prognosis of ischemic stroke has not yet been studied. The aim of this study was to investigate the association of serum Dkk-3 levels with prognosis in patients with ischemic stroke using data from the CATIS (China Antihypertensive Trial in Acute Ischemic Stroke).

## Methods

### Study design and patients

The data that support the findings of this study are available from the corresponding author upon reasonable request. This study was conducted among the ischemic stroke patients from the CATIS, a multicenter randomized clinical trial conducted in 26 hospitals across China from August 2009 to August 2013. Details on the study design and major results of the CATIS have been reported previously [12]. In brief, a total of 4071 patients aged ≥ 22 years who had ischemic stroke confirmed by computed tomography or magnetic resonance imaging of the brain within 48 h of symptom onset and with an elevated systolic blood pressure (BP) between 140 and 220 mm Hg were recruited. Patients with a systolic BP ≥ 220 or diastolic BP ≥ 120 mm Hg, severe heart failure, acute myocardial infarction or unstable angina, atrial fibrillation, aortic dissection, cerebrovascular stenosis (≥ 70%), or resistant hypertension; those in a deep coma; and those treated with intravenous thrombolytic therapy were excluded from CATIS. In the present study, we further excluded 727 participants because they did not offer blood samples, or some collected samples were hemolyzed in storage or transport. Finally, a total of 3344 patients were included in this analysis. Most baseline characteristics were balanced between patients who were assayed for serum Dkk-3 and those not assayed (Additional file 1: Table S1), indicating that those assayed basically represented all participants in the CATIS study.

The CATIS trial was registered at ClinicalTrials.gov (NCT01840072). This study was approved by the Institutional Review Boards or Ethical Committees at Soochow University in China, Tulane University in the USA, and all participating hospitals. Written consent was obtained from all study participants or their immediate family members.

### Data collection

Baseline data on demographic characteristics, lifestyle risk factors, medical history, and clinical features were collected at the time of enrollment. Stroke severity was assessed using the National Institutes of Health Stroke Scale (NIHSS) by trained neurologists at baseline [13]. Ischemic stroke was classified as large artery atherosclerosis (thrombotic), cardiac embolism (embolic), and small artery occlusion lacunae (lacunar) according to the symptoms and imaging data of the patients [14]. Three BP measurements were obtained at baseline according to a standard protocol adapted from procedures recommended by the American Heart Association [15]. Blood glucose (normal range < 7.0 mmol/L), total cholesterol (normal range < 6.22 mmol/L), triglyceride (normal range < 2.26 mmol/L), low-density lipoprotein cholesterol (normal range < 4.14 mmol/L), high-density lipoprotein cholesterol (normal range ≥ 1.04 mmol/L), creatinine (normal range women < 120 μmol/L; men < 136 μmol/L), and uric acid (normal range women 155–357 μmol/L; men 208–428 μmol/L) were measured for all enrolled patients in each participating hospital at baseline. Dyslipidemia was defined as total cholesterol ≥ 6.22 mmol/L or triglyceride ≥ 2.26 mmol/L or low-density lipoprotein cholesterol ≥ 4.14 mmol/L or high-density lipoprotein cholesterol < 1.04 mmol/L or self-reported history of physician-diagnosed dyslipidemia according to Chinese guidelines on the prevention and treatment of dyslipidemia [16].

### Serum Dkk-3 measurement

Fasting blood samples were collected after at least 8 h of fasting within 24 h of hospital admission. All serum samples were separated and immediately frozen at − 80 °C until tested. Serum Dkk-3 concentrations were measured centrally at Soochow University with a commercially available ELISA kit (Catalog: DY1118; R&D Systems, Inc, Minneapolis). Intra- and inter-assay coefficients of variation were < 4.6% and 6.6%, respectively. Laboratory technicians who performed these measurements were blinded to the clinical characteristics and outcomes of the study participants.

### Outcome assessment

The participants were followed up in person at 3 months after ischemic stroke by trained neurologists unaware of treatment assignment. The primary outcome was a combination of death and vascular events (i.e., vascular deaths, recurrent nonfatal stroke, nonfatal myocardial infarction, hospitalized and treated angina, hospitalized and treated congestive heart failure, and hospitalized and treated peripheral arterial disease). Secondary outcomes were separately death and vascular events. Death certificates were obtained for deceased patients, and hospital data were abstracted for all vascular events. The study outcome assessment committee, blinded to treatment assignment, reviewed and adjudicated vascular events based on the criteria established in the ALLHAT (Antihypertensive and Lipid-Lowering Treatment to Prevent Heart Attack Trial).

### Statistical analysis

All participants were categorized into five groups according to the quintiles of serum Dkk-3 concentrations. We presented baseline characteristics of the study participants as mean ± standard deviation or median (interquartile range) for continuous variables and as frequency (percentage) for categorical variables. Baseline characteristics among different quintiles of serum Dkk-3 were compared by analysis of variance or the Kruskal-Wallis test for continuous variables and *χ*^2^ test for categorical variables. The cumulative incidence risks of clinical outcomes across baseline serum Dkk-3 quintiles were estimated using the Kaplan-Meier curves and compared by log-rank tests. Cox proportional hazards regression analyses were used to assess the association between serum Dkk-3 and clinical outcomes of ischemic stroke. With the third quintile of serum Dkk-3 as a reference, hazard ratios (HRs) and 95% confidence intervals (CIs) were calculated for the other four quintiles of serum Dkk-3. The covariates included in the multivariable model were age, sex, time from onset to hospitalization, current smoking, alcohol consumption, dyslipidemia, body mass index, blood glucose, diastolic BP, creatinine, high sensitivity C-reactive protein, baseline NIHSS score, history of hypertension, history of coronary heart disease, history of diabetes mellitus, family history of stroke, ischemic stroke subtypes, and receiving immediate BP reduction. We further evaluated the pattern of the association between serum Dkk-3 and primary outcome using a Cox proportional hazards regression model with restricted cubic splines for serum Dkk-3 adjusting for aforementioned covariates, with 5 knots placed at the 10th, 30th, 50th, 70th, and 90th percentiles of Dkk-3 [17].

In the secondary analyses, we conducted subgroup analyses in Cox proportional hazards regression models to assess the robustness of the association between serum Dkk-3 levels and prognosis of ischemic stroke. Interactions between serum Dkk-3 and subgroup variables on the primary outcome were tested in the models with interaction terms by the likelihood ratio test, adjusting for the aforementioned covariates unless the variable was used as a subgroup variable. Net reclassification improvement (NRI) and integrated discrimination improvement (IDI) were two statistical indexes to assess improvement in model performance accomplished by adding new markers [18]. We constructed a conventional model (only including aforementioned covariates) and a new model (including aforementioned covariates and serum Dkk-3) by Cox proportional hazards regression model. To evaluate the incremental prognostic value of serum Dkk-3 beyond conventional risk factors, we calculated NRI and IDI through comparing these two models. All *p* values were two tailed, and a significance level of 0.05 was used. Statistical analysis was conducted with SAS statistical software (version 9.4, Cary, NC).

## Results

### Baseline characteristics of study participants

There were 3344 patients (2133 men and 1211 women) with a mean age of 62.3 years included in the present study. The median serum Dkk-3 level was 60.77 ng/mL (interquartile range 49.22–75.87 ng/mL). Compared with participants with lower serum Dkk-3 levels, those with higher Dkk-3 levels were more likely to be older and male; have higher creatinine; have higher prevalence of history of coronary heart disease and lacunar stroke; have lower diastolic BP, body mass index, total cholesterol, triglyceride, and blood glucose; and have lower prevalence of alcohol drinking, family history of stroke, and thrombotic stroke (the analysis of variance or Kruskal-Wallis test for continuous variables and *χ*^2^ test for categorical variables reveal *p* < 0.05; Table 1).

**Table 1:** Baseline characteristics of participants according to the quintiles of serum Dkk-3

Characteristics^*^	Dkk-3, ng/mL					*p* value^‡^
	< 46.57	46.57–55.77	55.77–65.69	65.69–79.80	≥ 79.80	
Number of patients	668	668	670	669	669	
Demographics						
Age, years	57.07 ± 9.90	59.64 ± 10.23	61.91 ± 10.12	64.85 ± 10.46	68.18 ± 10.06	< 0.001
Male	400 (59.88)	437 (65.42)	454 (67.76)	427 (63.83)	415 (62.03)	0.031
Current cigarette smoking	243 (36.38)	264 (39.52)	263 (39.25)	225 (33.63)	227 (33.93)	0.059
Current alcohol drinking	241 (36.08)	254 (38.02)	225 (33.58)	186 (27.80)	142 (21.23)	< 0.001
Medical history						
History of hypertension	525 (78.59)	535 (80.09)	522 (77.91)	519 (77.58)	526 (78.62)	0.830
History of coronary heart disease	40 (5.99)	55 (8.23)	65 (9.70)	83 (12.41)	101 (15.10)	< 0.001
History of diabetes mellitus	132 (19.76)	109 (16.32)	109 (16.27)	112 (16.74)	121 (18.09)	0.387
Family history of stroke	122 (18.26)	146 (21.86)	134 (20.00)	112 (16.74)	99 (14.80)	0.009
Clinical features						
Time from onset to hospitalization, h	12.0 (5.0–24.0)	11.8 (5.0–24.0)	10.0 (4.0–24.0)	10.0 (4.0–24.0)	10.0 (4.0–24.0)	0.427
Systolic BP, mm Hg	166.55 ± 17.14	167.29 ± 17.04	165.60 ± 17.13	165.79 ± 16.44	166.92 ± 16.64	0.296
Diastolic BP, mm Hg	98.65 ± 11.08	97.60 ± 10.90	96.96 ± 11.18	95.48 ± 10.28	94.69 ± 11.31	< 0.001
Body mass index, kg/m^2^	25.67 ± 3.08	25.14 ± 3.10	24.87 ± 2.78	24.70 ± 3.11	24.24 ± 3.16	< 0.001
Total cholesterol, mmol/L	5.10 (4.41–5.90)	5.06 (4.35–5.72)	4.92 (4.28–5.65)	5.01 (4.29–5.73)	4.90 (4.17–5.67)	0.005
Triglyceride, mmol/L	1.68 (1.20–2.46)	1.53 (1.10–2.29)	1.47 (1.02–2.03)	1.38 (0.99–2.00)	1.30 (0.94–1.91)	< 0.001
Low-density lipoprotein cholesterol, mmol/L	2.90 (2.36–3.47)	2.91 (2.34–3.53)	2.89 (2.27–3.53)	2.90 (2.26–3.43)	2.82 (2.26–3.50)	0.570
High-density lipoprotein cholesterol, mmol/L	1.20 (1.02–1.48)	1.23 (1.04–1.48)	1.22 (1.02–1.49)	1.22 (1.04–1.48)	1.27 (1.06–1.53)	0.099
Blood glucose, mmol/L	6.00 (5.20–7.40)	5.80 (5.10–7.41)	5.70 (5.00–7.03)	5.71 (5.08–7.10)	5.60 (4.90–7.00)	< 0.001
Creatinine, μmol/L	65.0 (54.0–76.8)	67.3 (56.0–78.0)	69.0 (59.0–81.0)	70.0 (60.0–83.6)	73.0 (61.0–90.9)	< 0.001
Uric acid, μmol/L	280.00 (228.65–352.65)	278.00 (230.30–336.10)	285.80 (236.00–349.00)	284.10 (233.30–336.00)	284.50 (231.15–354.00)	0.724
High sensitivity C-reactive protein, mg/L	2.00 (0.80–4.30)	1.90 (0.80–4.40)	1.70 (0.60–4.50)	1.90 (0.70–4.90)	1.90 (0.70–5.70)	0.402
Baseline NIHSS score	4.0 (3.0–8.0)	4.0 (2.0–7.0)	4.0 (2.0–8.0)	4.0 (2.0–7.0)	5.0 (3.0–8.0)	0.385
Ischemic stroke subtype^†^						
Thrombotic	544 (81.44)	518 (77.54)	509 (75.97)	508 (75.93)	483 (72.20)	0.002
Embolic	25 (3.74)	24 (3.59)	35 (5.22)	38 (5.68)	42 (6.28)	0.088
Lacunar	109 (16.32)	140 (20.96)	149 (22.24)	144 (21.52)	159 (23.77)	0.013
Receiving immediate BP reduction	341 (51.05)	334 (50.00)	329 (49.10)	320 (47.83)	344 (51.42)	0.683

*BP* blood pressure, *Dkk-3* dickkopf-3, *NIHSS* National Institutes of Health Stroke Scale

*Continuous variables are expressed as mean ± standard deviation, or as median (interquartile range). Categorical variables are expressed as frequency (percentage)

^†^Ten patients with both thrombotic and embolic subtypes, 76 patients with thrombotic and lacunar subtypes, 5 patients with embolic and lacunar subtypes, and 1 patient with all 3 subtypes

^‡^*p* values were based on the analysis of variance or Kruskal-Wallis test for continuous variables and *χ*^2^ test for categorical variables

### Serum Dkk-3 levels and clinical outcomes

**Fig. 1:**
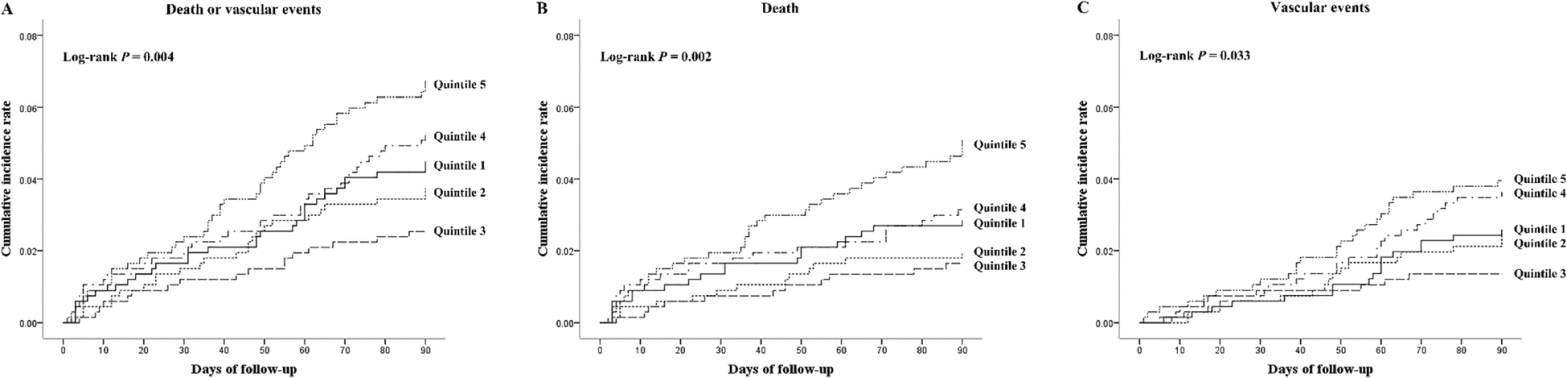
Cumulative incidence curves of clinical outcomes according to the quintiles of serum Dkk-3 at baseline. **a** Primary outcome (death or vascular events). **b** Death. **c** Vascular events. *p* values were based on the log-rank test

Within 3 months after ischemic stroke onset, a total of 152 patients (4.55%) experienced the primary outcome (a composite outcome of death and vascular events). Among them, 60 were death, 54 were vascular events, and 38 were both death and vascular events. Figure 1 shows the Kaplan-Meier curves for cumulative incidences of primary outcome, death, and vascular events across serum Dkk-3 quintiles. The cumulative incidence rates of primary outcome within 3 months among ischemic stroke patients in five quintiles of serum Dkk-3 (from low to high) were 4.49%, 3.74%, 2.54%, 5.23%, and 6.73%, respectively. The patients in the third quintile of serum Dkk-3 had the lowest cumulative incidence rate of primary outcome (log-rank *p* = 0.004) compared to those in the other four quintiles of serum Dkk-3. Similarly, the lowest cumulative incidence rates of death (log-rank *p* = 0.002) and vascular events (log-rank *p* = 0.033) within 3 months after ischemic stroke were also observed among patients in the third quintile of serum Dkk-3.

**Fig. 2:**
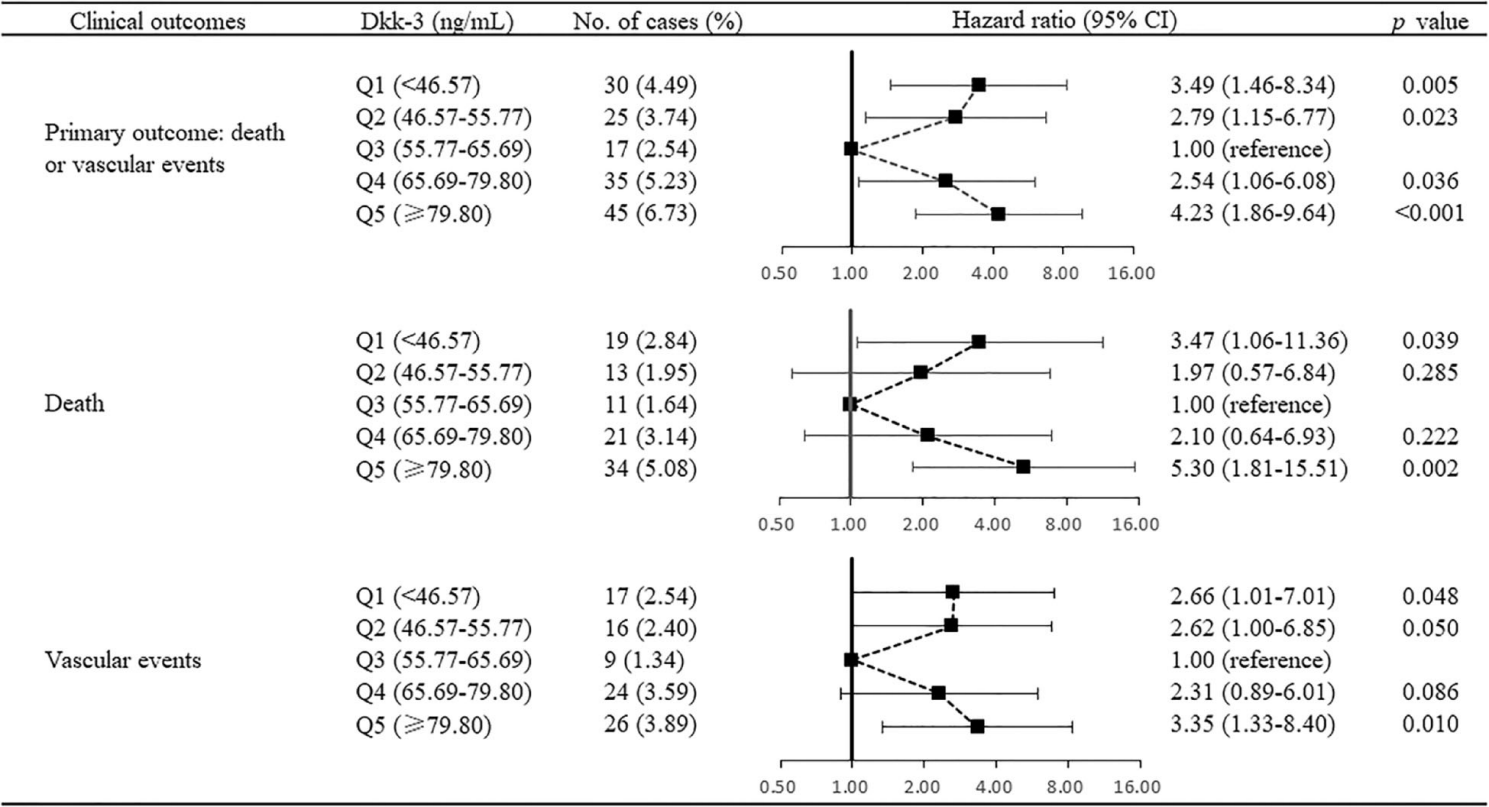
Hazard ratios (95% confidence intervals) of clinical outcomes according to the quintiles of serum Dkk-3 at baseline. Cox proportional hazards regression analyses were used to assess the association between serum Dkk-3 and clinical outcomes of ischemic stroke. Multivariate adjusted hazard ratios were estimated with adjustment for age, sex, time from onset to hospitalization, current smoking, alcohol consumption, dyslipidemia, body mass index, blood glucose, diastolic blood pressure, creatinine, high sensitivity C-reactive protein, baseline NIHSS score, history of hypertension, history of coronary heart disease, history of diabetes mellitus, family history of stroke, ischemic stroke subtypes, and receiving immediate blood pressure reduction. *p* values were based on the analysis of maximum likelihood estimates

**Fig. 3:**
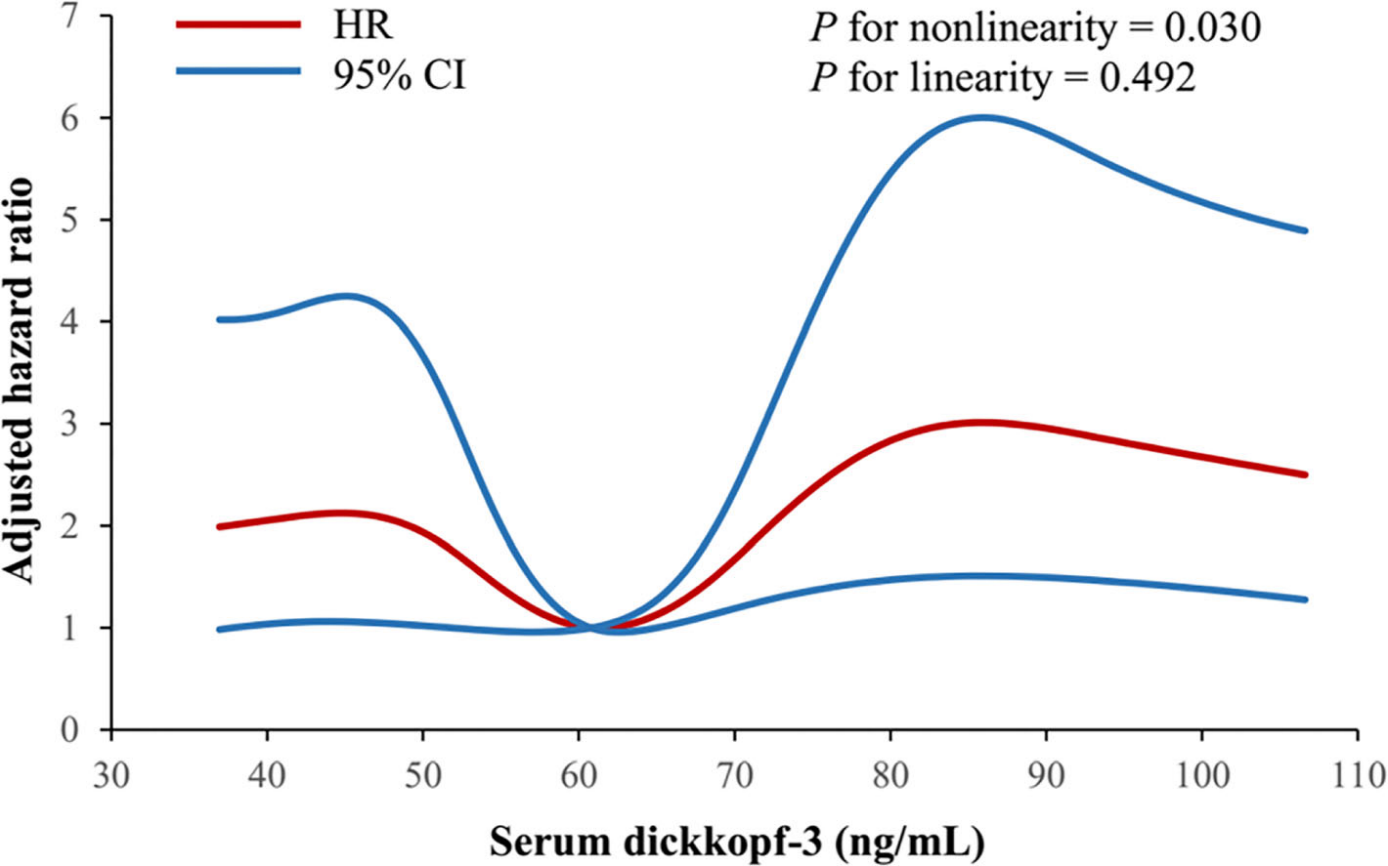
U-shaped association between serum Dkk-3 and the risk of primary outcome (death or vascular events) among patients with ischemic stroke. Hazard ratios (HRs) and 95% confidence intervals were derived from a Cox proportional hazards regression model with restricted cubic splines, with knots placed at the 10th, 30th, 50th, 70th, and 90th percentiles of the distribution of serum Dkk-3. Reference point was the median value of serum Dkk-3 (60.78 ng/mL). HRs were adjusted for age, sex, time from onset to hospitalization, current smoking, alcohol consumption, dyslipidemia, body mass index, blood glucose, diastolic blood pressure, creatinine, high sensitivity C-reactive protein, baseline NIHSS score, history of hypertension, history of coronary heart disease, history of diabetes mellitus, family history of stroke, ischemic stroke subtypes, and receiving immediate blood pressure reduction. The red line indicated HR, and the blue lines indicated 95% CI. *p* values were based on the likelihood ratio test

We used Cox proportional hazards regression models to assess the association of serum Dkk-3 quintiles at baseline with primary outcome, death, and vascular events within 3 months after ischemic stroke (Fig. 2). Compared with the third quintile of serum Dkk-3, the adjusted HRs (95% CIs) of the first and fifth quintile were 3.49 (1.46–8.34) and 4.23 (1.86–9.64) for primary outcome, 3.47 (1.06–11.36) and 5.30 (1.81–15.51) for death, and 2.66 (1.01–7.01) and 3.35 (1.33–8.40) for vascular events, respectively, indicating that both low and high serum Dkk-3 levels were associated with increased risk of poor prognosis. We further used a Cox proportional hazards regression model with restricted cubic splines to evaluate the pattern of association between serum Dkk-3 and primary outcome. As shown in Fig. 3, we observed a U-shaped association of serum Dkk-3 with the risk of primary outcome (the likelihood ratio test reveals *p* for nonlinearity = 0.030; the likelihood ratio test reveals *p* for linearity = 0.492).

### Subgroup analyses

We conducted subgroup analyses in the Cox proportional hazards regression models to examine the potential effect modification by age, sex, body mass index, baseline diastolic BP, baseline NIHSS score, cigarette smoking, alcohol consumption, and receiving immediate BP reduction medication on the association of serum Dkk-3 with primary outcome. The modest U-shaped associations between serum Dkk-3 and primary outcome were observed in all subgroups and reached significant levels in several subgroups. Moreover, statistical tests for the interactions between serum Dkk-3 and these interesting factors on primary outcome were not significant (the likelihood ratio test reveals all *p* value for interaction > 0.05; Additional file 1: Table S2).

### Incremental prognostic value of serum Dkk-3

We examined whether adding serum Dkk-3 to a Cox proportional hazards regression model consisting of conventional risk factors improved the risk prediction of adverse clinical outcomes after ischemic stroke. As shown in Table 2, the addition of aberrant serum Dkk-3 to conventional risk factors significantly improved predictive power for primary outcome (NRI 28.44%, the asymptotic test reveals *p* < 0.001; IDI 0.48%, the asymptotic test reveals *p* = 0.001).

**Table 2:** Reclassification and discrimination statistics for adverse clinical outcomes by serum Dkk-3 among ischemic stroke patients

Clinical outcomes	Model	Continuous NRI, %		IDI, %	
		Estimate (95% CI)	*p* value^†^	Estimate (95% CI)	*p* value^†^
Death or vascular events	Conventional model				
	Conventional model + aberrant serum Dkk-3 levels^*^	28.44 (17.99–38.88)	< 0.001	0.48 (0.19–0.77)	0.001
Death	Conventional model				
	Conventional model + aberrant serum Dkk-3 levels^*^	28.81 (15.96–41.66)	< 0.001	0.40 (− 0.33–1.12)	0.282
Vascular events	Conventional model				
	Conventional model + aberrant serum Dkk-3 levels^*^	24.95 (11.60–38.29)	< 0.001	0.30 (0.17–0.44)	< 0.001

Conventional Cox proportional hazards regression model included age, sex, time from onset to hospitalization, current smoking, alcohol consumption, dyslipidemia, body mass index, blood glucose, diastolic blood pressure, creatinine, high sensitivity C-reactive protein, baseline NIHSS score, history of hypertension, history of coronary heart disease, history of diabetes mellitus, family history of stroke, ischemic stroke subtypes, and receiving immediate blood pressure reduction

*CI* confidence interval, *Dkk-3* dickkopf-3, *IDI* integrated discrimination improvement, *NRI* net reclassification improvement

*The aberrant serum Dkk-3 levels were defined as the low or high levels of serum Dkk-3 (first, second, fourth, and fifth quintile of Dkk-3)

^†^*p* values were based on the asymptotic test

## Discussion

To our knowledge, this is the first study to investigate the association of serum Dkk-3 with death and vascular events after ischemic stroke. In this large-sample multicenter study of ischemic stroke patients, we found a U-shaped association between serum Dkk-3 and the risk of death and vascular events after adjustment for potential confounders. Namely, both low and high serum Dkk-3 levels at baseline were associated with increased risk of poor prognosis, with minimum risk observed in the third quintile of serum Dkk-3. Subgroup analyses further confirmed these findings. Moreover, the addition of serum Dkk-3 to conventional prognostic factors could significantly improve the risk prediction for death and vascular events after ischemic stroke, as evidenced by NRI and IDI. These findings indicate that serum Dkk-3 may have a special effect on the prognosis of ischemic stroke, that is, both too low and too high serum Dkk-3 levels are harmful to the prognosis of ischemic stroke. Further studies from various populations are needed to replicate our findings.

Dkk-3 has been demonstrated to be closely involved in the development and progression of cardiovascular disease [8, 9]. Several studies showed that Dkk-3 overexpression could suppress endothelial cell growth and angiogenesis [19, 20]. High expression of Dkk-3 in human coronary artery plaques could accelerate the atherosclerotic process and promoted plaque accumulation, while reduction of Dkk-3 attenuated the atherosclerotic lesion burden through decreasing the size of atherosclerotic lesions and increasing the stability of plaques [8]. In contrast, as an important regulator of cell fate determination [10], Dkk-3 had a certain role in the protection against atherosclerosis involving endothelial migration and repair. Dkk-3 could promote re-endothelialization and reduced neointima formation after atherosclerosis or artery injury, and the absence of Dkk-3 was also related to incident carotid atherosclerosis and stenosis during 5-year follow-up [9]. In addition, some other studies reported that Dkk-3 was a cardioprotective regulator and could protect against cardiac dysfunction following myocardial infarction [21, 22]. All these above studies seem to indicate that Dkk-3 may have a special role (both protective and harmful effects) in the cardiovascular disease, but no such evidence is available for ischemic stroke.

Herein, we conducted a multicenter study based on the CATIS randomized clinical trial with a large sample size, standardized protocols, and rigid quality control procedures. In addition, comprehensive information about potential confounders was collected and controlled in the multivariate models. Such methodological advantages enable us to draw more accurate conclusions for the association between serum Dkk-3 and clinical outcomes after ischemic stroke. In the present study, we found a U-shaped association between serum Dkk-3 and poor prognosis, which was concordant with the observed complex effects of Dkk-3 on the atherosclerosis. The similar complex effects on ischemic stroke were previously reported for other biochemical indexes such as blood glucose and hemoglobin [23–25]. For example, hyperglycemia is an established risk factor for the poor prognosis of ischemic stroke while hypoglycemia is also associated with brain damage and poor outcomes, namely, there is also a U-shaped association between blood glucose and adverse clinical outcomes after ischemic stroke [23, 24]. Given that age was a major predisposing factor for prognosis of ischemic stroke and serum Dkk-3 level was positively related to age in this study and the previous study [9], we adjusted age in the multivariable Cox proportional hazards regression model and performed further subgroup analysis stratified by age to weaken or even eliminate the confounding effects of age on the association between serum Dkk-3 and prognosis of ischemic stroke. We found that the significant association of serum Dkk-3 with death and vascular events was independent of age, and there was no modified effect of age on the association of serum Dkk-3 with death and vascular events, suggesting that serum Dkk-3 might have additional prognostic value when age was considered. The present study provided valuable evidence for the important role of serum Dkk-3 in the pathogenesis of ischemic stroke.

Our study has important clinical implications for better understanding the effects of Dkk-3 in pathological process after ischemic stroke onset. From findings of this study, serum Dkk-3 might be useful in the risk stratification of prognosis of ischemic stroke and could assist the selection of high-risk patients for aggressive monitoring and therapeutic interventions. Further studies from other samples of ischemic stroke patients are needed to validate the effects or prognostic value of serum Dkk-3 after ischemic stroke. In addition, considering the existence of upper and lower bounds for serum Dkk-3 like other clinical biochemical markers, it is of clinical interest to determine the optimal concentration of serum Dkk-3 in the further studies.

Several biological mechanisms may underlie the observed U-shaped association of serum Dkk-3 with death and vascular events after ischemic stroke. On the one hand, Wnt/β-catenin signaling pathway is involved in the regulation of survival and proliferation of vascular cells [26]. As an antagonist of Wnt/β-catenin pathway [6, 7, 27], the overexpression of Dkk-3 could accelerate the atherosclerotic process and resulted in detrimental outcomes of atherosclerosis through inhibiting this pathway [8]. The high expression of Dkk-3 also appears to downregulate the level of vascular endothelial growth factor [7, 20] and then suppresses endothelial cell growth and angiogenesis [19, 20]. On the other hand, besides the adverse effects of Dkk-3 overexpression, it is necessary to maintain a certain level of Dkk-3 in tissues or blood in normal physiological process [10]. Dkk-3 has a decisive function in the vascular cell differentiation [28], and it can induce endothelial migration and repair through activating JNK pathway [9]. In addition, Dkk-3 is proposed to be involved in maintaining the endothelium integrity, and the endothelium is apparently dysfunctional in the mice with deficiency of Dkk-3 [9]. In view of these biological mechanisms, both low and high serum Dkk-3 levels might induce increased risks of death and vascular events after ischemic stroke.

Our study has several limitations. First, this study is not a specially designed study for the association between serum Dkk-3 and prognosis of ischemic stroke, but an observational study based on the participants from CATIS. Therefore, there may exist a selection bias. However, baseline characteristics of participants in this study were similar to those from the China National Stroke Registry [29], suggesting that the selection bias might be minimal. Further studies from other samples of ischemic stroke patients are needed to validate our findings. Second, we did not conduct serial measurements of serum Dkk-3 levels after ischemic stroke onset, so we were unable to examine the association between changes of serum Dkk-3 and prognosis of ischemic stroke. Finally, our study was an exploratory study, so we were unable to assess the medical reference ranges of serum Dkk-3. Further studies are needed to determine the optimal concentration of serum Dkk-3.

## Conclusions

Both low and high serum Dkk-3 levels are associated with death and vascular events within 3 months after ischemic stroke, with minimum risk observed in the third quintile of serum Dkk-3, indicating that serum Dkk-3 may have a special effect on the prognosis of ischemic stroke. We also found that serum Dkk-3 might be a prognostic biomarker for ischemic stroke. Further studies are needed to replicate our findings and to determine the optimal levels of serum Dkk-3.

## Supplementary information

**Additional file 1: Table S1.** Baseline characteristics between the serum dickkopf-3 assayed and not-assayed groups. **Table S2.** Subgroup analyses of the association between serum Dkk-3 and primary outcome (death or vascular events).

## Data Availability

The datasets used and/or analyzed during the current study are available from the corresponding author on reasonable request

## Abbreviations

ALLHAT: Antihypertensive and Lipid-Lowering Treatment to Prevent Heart Attack Trial
BP: Blood pressure
CATIS: China Antihypertensive Trial in Acute Ischemic Stroke
CI: Confidence interval
Dkk-3: Dickkopf-3
HR: Hazard ratio
IDI: Integrated discrimination improvement
NIHSS: National Institutes of Health Stroke Scale
NRI: Net reclassification improvement
