# Supplementary material for: Serum dickkopf-3 is associated with death and vascular events after ischemic stroke: an observational study from CATIS
Source: J Neuroinflammation. 2020 Jan 9;17:12. doi: 10.1186/s12974-019-1680-4 (PMC6953290; doi:10.1186/s12974-019-1680-4)
Supplement: Supplementary file 1 — Additional file 1: Table S1. Baseline characteristics between the serum dickkopf-3 assayed and not-assayed groups. Table S2. Subgroup analyses of the association between serum Dkk-3 and primary outcome (death or vascular events). [file 12974_2019_1680_MOESM1_ESM.docx]

**SUPPLEMENTAL MATERIAL**

**Serum dickkopf-3 is associated with death and vascular events after ischemic stroke: A prospective study from CATIS**

**Supplemental Table 1.** Baseline characteristics between the serum dickkopf-3 assayed and not-assayed groups

| Characteristics | Assayed | Not-assayed | *p* value^‡^ |
| --- | --- | --- | --- |
| Number of patients | 3344 | 727 |  |
| **Demographics** |  |  |  |
| Age, years | 62.33 ± 10.87 | 63.24 ± 11.01 | 0.042 |
| Male | 2133 (63.79) | 471 (64.79) | 0.610 |
| Current cigarette smoking | 1222 (36.54) | 263 (36.18) | 0.852 |
| Current alcohol drinking | 1048 (31.34) | 205 (28.20) | 0.096 |
| **Medical history** |  |  |  |
| History of hypertension | 2627 (78.56) | 582 (80.06) | 0.371 |
| History of coronary heart disease | 344 (10.29) | 100 (13.76) | 0.007 |
| History of diabetes mellitus | 583 (17.43) | 136 (18.71) | 0.415 |
| Family history of stroke | 613 (18.33) | 140 (19.26) | 0.560 |
| **Clinical features** |  |  |  |
| Time from onset to hospitalization, h | 10.0 (4.5-24.0) | 10.7 (5.0-24.0) | 0.315 |
| Systolic BP, mm Hg | 166.43 ± 16.88 | 164.79 ± 16.94 | 0.018 |
| Diastolic BP, mm Hg | 96.68 ± 11.04 | 96.68 ± 11.38 | 0.990 |
| Body mass index, kg/m^2^ | 24.93 ± 3.08 | 25.07 ± 3.36 | 0.356 |
| Dyslipidemia | 1848 (55.26) | 404 (55.57) | 0.880 |
| Blood glucose, mmol/L | 5.80 (5.09-7.20) | 5.80 (5.10-7.36) | 0.334 |
| Creatinine, μmol/L | 69.00 (58.00-81.00) | 70.00 (58.65-84.05) | 0.053 |
| High sensitivity C-reactive protein, mg/L | 1.90 (0.70-4.70) | 1.90 (0.70-8.00) | 0.056 |
| Baseline NIHSS score | 4.0 (2.0-8.0) | 4.0 (2.0-7.0) | 0.052 |
| **Ischemic stroke subtype^†^** |  |  |  |
| Thrombotic | 2562 (76.61) | 608 (83.63) | <0.001 |
| Embolic | 164 (4.90) | 38 (5.23) | 0.717 |
| Lacunar | 701 (20.96) | 101 (13.89) | <0.001 |
| Receiving immediate blood pressure reduction | 1668 (49.88) | 370 (50.89) | 0.620 |

BP = blood pressure; NIHSS = National Institutes of Health Stroke Scale.

* Continuous variables are expressed as mean ± standard deviation, or as median (interquartile range). Categorical variables are expressed as frequency (percentage).

† Twelve patients with both thrombotic and embolic subtypes; 93 patients with thrombotic and lacunar subtypes; 6 patients with embolic and lacunar subtypes; 1 patient with all 3 subtypes.

‡ *p* values were based on the Student's t-test or Wilcoxon rank-sum test for continuous variables and χ^2^ test for categorical variables.

**Supplemental Table 2.** Subgroup analyses of the association between serum Dkk-3 and primary outcome (death or vascular events).

| Subgroup | Dkk-3, ng/mL | | | | | *p*_interaction_ |
| --- | --- | --- | --- | --- | --- | --- |
|  | <46.57 | 46.57-55.77 | 55.77-65.69 | 65.69-79.80 | ≥79.80 |  |
| Age, years |  |  |  |  |  | 0.577 |
| <65 | 1.56 (0.48-5.09) | 1.70 (0.51-5.73) | 1.00 | 2.90 (0.88-9.54) | 2.24 (0.59-8.48) |  |
| ≥65 | 8.55 (2.32-31.50) | 4.25 (1.12-16.08) | 1.00 | 3.18 (0.86-11.69) | 7.34 (2.18-24.71) |  |
| Sex |  |  |  |  |  | 0.813 |
| Men | 2.65 (0.90-7.86) | 3.49 (1.24-9.84) | 1.00 | 2.66 (0.91-7.77) | 4.15 (1.53-11.24) |  |
| Women | 6.30 (1.30-30.55) | 1.82 (0.30-11.07) | 1.00 | 3.52 (0.74-16.84) | 6.21 (1.34-28.79) |  |
| Body mass index, kg/m^2^ |  |  |  |  |  | 0.431 |
| <24 | 2.09 (0.93-4.72) | 1.24 (0.54-2.85) | 1.00 | 1.09 (0.49-2.46) | 1.33 (0.62-2.84) |  |
| ≥24 | 4.47 (1.25-16.04) | 3.66 (1.00-13.38) | 1.00 | 3.50 (0.97-12.69) | 5.38 (1.55-18.64) |  |
| Baseline diastolic BP, mmHg |  |  |  |  |  | 0.378 |
| <100 | 2.72 (0.80-9.21) | 3.06 (0.94-9.96) | 1.00 | 2.72 (0.86-8.63) | 4.45 (1.49-13.32) |  |
| ≥100 | 5.04 (1.36-18.61) | 2.31 (0.59-9.11) | 1.00 | 2.04 (0.50-8.29) | 5.78 (1.58-21.20) |  |
| Baseline NIHSS score |  |  |  |  |  | 0.488 |
| <4 | 5.16 (0.58-45.53) | 3.70 (0.40-33.95) | 1.00 | 3.77 (0.42-33.56) | 12.98 (1.60-105.18) |  |
| ≥4 | 3.61 (1.41-9.25) | 2.39 (0.90-6.33) | 1.00 | 2.30 (0.89-5.97) | 3.22 (1.30-8.00) |  |
| Cigarette smoking |  |  |  |  |  | 0.312 |
| No | 4.76 (1.54-14.75) | 3.51 (1.12-11.05) | 1.00 | 2.87 (0.93-8.81) | 4.83 (1.63-14.31) |  |
| Yes | 1.98 (0.48-8.10) | 1.60 (0.37-6.86) | 1.00 | 2.18 (0.51-9.26) | 3.93 (1.09-14.17) |  |
| Alcohol consumption |  |  |  |  |  | 0.246 |
| No | 3.27 (1.23-8.65) | 2.37 (0.89-6.37) | 1.00 | 2.04 (0.79-5.30) | 3.15 (1.28-7.76) |  |
| Yes | 7.38 (0.88-62.03) | 5.80 (0.66-50.71) | 1.00 | 7.10 (0.77-65.88) | 15.35 (1.87-125.64) |  |
| Receiving immediate BP reduction |  |  |  |  |  | 0.979 |
| No | 10.40 (1.30-83.02) | 10.43 (1.31-83.09) | 1.00 | 11.34 (1.45-88.46) | 14.97 (1.94-115.26) |  |
| Yes | 2.78 (0.99-7.83) | 1.75 (0.59-5.24) | 1.00 | 1.27 (0.43-3.78) | 2.25 (0.86-5.93) |  |

In the multivariate Cox proportional hazards regression model, confounding factors such as age, sex, time from onset to hospitalization, current smoking, alcohol consumption, dyslipidemia, body mass index, blood glucose, diastolic blood pressure, creatinine, high sensitivity C-reactive protein, baseline NIHSS score, history of hypertension, history of coronary heart disease, history of diabetes mellitus, family history of stroke, ischemic stroke subtypes, and receiving immediate blood pressure reduction were included unless the variable was used as a subgroup variable. *p*_interaction_ values were based on the likelihood ratio test.
